# Correction : Selected articles from the annual update in intensive care and emergency medicine 2020

**DOI:** 10.1186/s13054-024-04856-9

**Published:** 2024-03-21

**Authors:** Critical Care

**Affiliations:** London, UK

**Correction: Crit Care** **24**, **104 (2020)**. 10.1186/s13054-020-2776-z; **Crit Care 24, 106 (2020)**. 10.1186/s13054-020-2777-y; **Crit Care 24, 102 (2020)**. 10.1186/s13054-020-2778-x; **Crit Care 24, 99 (2020)**. 10.1186/s13054-020-2779-9; **Crit Care 24, 105 (2020)**. 10.1186/s13054-020-2780-3; **Crit Care 24, 100 (2020)**. 10.1186/s13054-020-2781-2; **Crit Care 24, 97 (2020)**. 10.1186/s13054-020-2782-1; **Crit Care 24, 103 (2020)**. 10.1186/s13054-020-2783-0; **Crit Care 24, 98 (2020)**. 10.1186/s13054-020-2784-z;** Crit Care 24, 101 (2020)**. 10.1186/s13054-020-2785-y.

The original publications of the following articles did not contain a license text in the published version. The correct license text is provided in this correction article, all listed articles have the same license. The original articles have been updated to correct this error.

## Correct license text

**Open Access** This article is licensed under a Creative Commons Attribution 4.0 International License, which permits use, sharing, adaptation, distribution and reproduction in any medium or format, as long as you give appropriate credit to the original author(s) and the source, provide a link to the Creative Commons licence, and indicate if changes were made. The images or other third party material in this article are included in the article’s Creative Commons licence, unless indicated otherwise in a credit line to the material. If material is not included in the article’s Creative Commons licence and your intended use is not permitted by statutory regulation or exceeds the permitted use, you will need to obtain permission directly from the copyright holder. To view a copy of this licence, visit http://creativecommons.org/licenses/by/4.0/. The Creative Commons Public Domain Dedication waiver (http://creativecommons.org/publicdomain/zero/1.0/) applies to the data made available in this article, unless otherwise stated in a credit line to the data.

## Affected articles

10.1186/s13054-020-2776-z

10.1186/s13054-020-2777-y

10.1186/s13054-020-2778-x

10.1186/s13054-020-2779-9

10.1186/s13054-020-2780-3

10.1186/s13054-020-2781-2

10.1186/s13054-020-2782-1

10.1186/s13054-020-2783-0

10.1186/s13054-020-2784-z

10.1186/s13054-020-2785-y

